# Supplementary material for: Eroded telomeres are rearranged in quiescent fission yeast cells through duplications of subtelomeric sequences
Source: Nat Commun. 2017 Nov 22;8:1684. doi: 10.1038/s41467-017-01894-6 (PMC5700057; doi:10.1038/s41467-017-01894-6)
Supplement: Supplementary file 1 — Supplementary Information [file 41467_2017_1894_MOESM1_ESM.pdf]

a

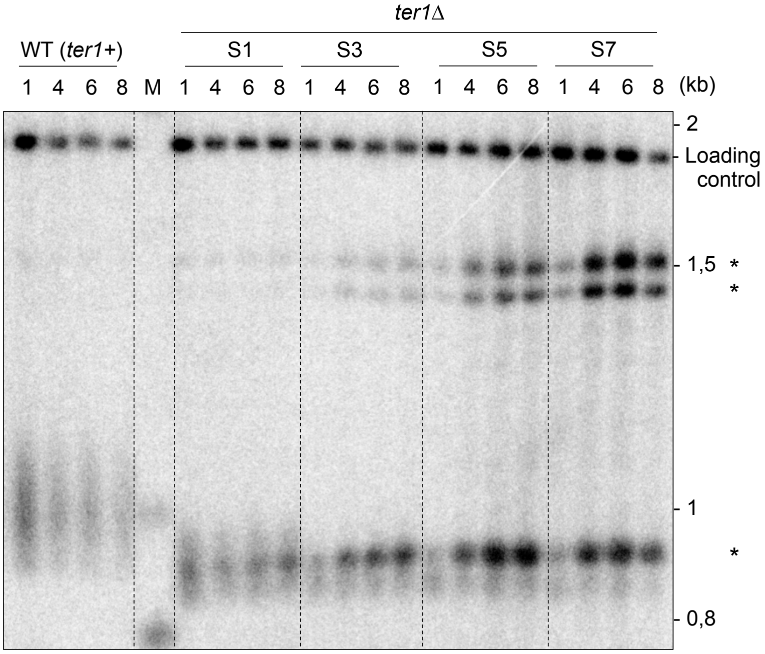

b

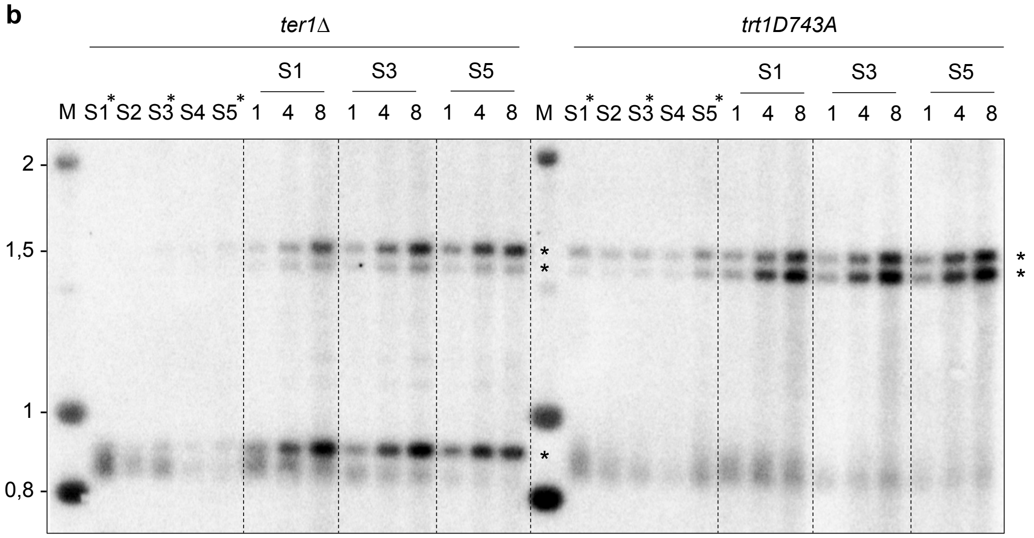

Supplementary Figure 1. **Eroded telomeres are rearranged in quiescence.**

a) Genomic DNA from quiescent *ter1*+ and *ter1*D cells was digested by *EcoR*I and analyzed by Southern blot with Telo/STE1 and chromosomal probes. 1, 4, 6, and 8 correspond to the number of days in G0 before harvesting. For *ter1*Δ cells, S1, S3, S5 and S7 indicate the number of days in senescence before cells were starved from nitrogen. STEEx are shown by asterisks

b) A senescence kinetics was performed from a strain expressing a catalytically dead version of the telomerase (*trt1*^{D743A}) after the loss of a plasmid bearing *trt1*+. At days 1, 3 and 5 of senescence, *trt1*^{D743A} cells were starved from nitrogen and kept for 8 days in quiescence. Cells were collected at different time points of the senescence kinetics (S1-S5) and for S1, S3, and S5 cells were starved from nitrogen and collected after 1, 4, and 8 days in quiescence. A *ter1*D strain was used as control. Genomic DNA was extracted, digested with *EcoR*I and analyzed by Southern blot with the STE1/Telo probe. STEEx was detected in the *trt1*^{D743A} strain according to its *Nsi*I pattern (Supplementary Figure 3) and are shown by asterisks

>pNSU64

TCAGATGCATAAAAAATTTGAAGTTGGTATGTATTGAGTGTGTTGGAGTACGGTAAGTATAATAGGGGTAAATAA
NsiI
AATGGGTAAAAAAAATTTTGAAATGTGTGGAAGTTGAGTATGTTGGAGTACATTAAGTAGATTACAGTTGTGGAG
p*NsiI* Fw
CGTACTATGGTAATGAAAATGAATGAAGAAAATGAAGTTGGGTTGAATTGAGCGTGGTAGGATTCATTATGTATG
ATTAGGAGATATAATGAGATATGGTGAATAAAAAGTTGAAATGTGTGGGCTTGAGTGCGTTAGGGTGCAGTAAGT
p*NsiI* Rev
AGAATAAAGGGGCGCAGTGTATTATGATAATTAAAATGGATGAAAAATTTGAAGTTCACTCAGTCATAATTAATT
p*ApaI* Fw
GGGTAACGGAGTAACAATATAGAATAAAGGGAATTTAGGAAGTGCGGTAAGTTGAATAAAGAAATAGAAATGAAA
HRS

TACGGTATTCATAAAAAAATAAATTTACTTAAGTTTTTTTCACAAATACAATGCCCCCACTATTGGGCCCCACC
ApaI
CGTCAGCCGAGCCGTACGGCGAGTATTCGTTAAACGATTTTGGAGAGAGAGAATGGATAATGGATGGAGGTAAGA
pApaI Rev
GAGGTATGAAAGAGTAGAAGATATAGAAGAGAAGAGAGAAGAAATGAGGAAGAAGTAAGATGAATAGAATAGAAG
AAACAGAGTAAGGAGAGTAAATAAATAAAGTAAGAGAGTAAAAGAAATAAGTAAAGTAAGAGGAAAGAAGATAAA
AGCAGAGGACTATATTGGAGTAGATTAAGTAGATTACAGTTGTGCAGCGTAGTATGATAATGAAGATAAAGAAAG
p*SwaI* Fw
ATAATTAAGCTGCGTTATTTATAAAAATTTAAATTTACTTAAGTTTTTTTCACATATACAATGCCCCCACTATTG
SwaI
GGCCCCACCCGTCAGCCGAGCCGTAAGGCGAGTATTCGTTAAACGTTTTTAGAGAGAGAGAAGGAAATGAAGGAG
p*SwaI* Rev
AGAAGGAAATGAAGGAGGAGAATAGGTAGAGTAGGTAGGGTATGGAAGAAAGAAGAGGAAGAAAGTAATGGAATA
GAATAAATAGAATAAATAGAATAAATAAAATAAATAAAATAAAAGAGTAAAAGAAAAGAAGATGAATGGATTAAA
AGGTGTTGGAGTAGATTAAGTAGAATACGAGGATGTAGGGTAGTGCAATAGTGAAGATGGACAAAAAGTTGAAGT
TCATGGAATTAGACTATGTTGGAATTCACTAATTGTAATACGGTGGTGTAGTGTGCATGGGTGAATAAAACGGAT
EcoRI
GAAAAATTTGAAGTTGATTTGAACTGAGTGTGTTAAAGTTCATTAAGTATAATACGGTGATGTAATGTACTATAG
TATTTAGGATAGATAAAAAATTTGAAGTTGTATGAATTGAGTGTGCTGGAATACGTTAAGTATAATACAGTGATG
TAGTGTACTATAATAATTAGGATGGGTAAAAAATTTGAAGTTGTATGAATTGAGTGTGTTATAGTTCATTAAGTA
TAATACGGTGATGTAGTGTACTATAGTATTTAGGATGGGTAAAAAATTGAAATGTGTGGAATTGAGTATGTTGAA
ATTCACTAAGTGTAATACTGTAGTGCAGCGTAATATGGTAATGGTAATGGATGAAAAATTTGAAGTTCACTCAGT
CATAATTAATTGGGTTACAAAGTAACAATGTAGAATAAGGAGCAATTAGAACGTACGGTAATATAGATAAAGAAA
GATAATTAAGTTGCGTTATTTATAAAAATTTAAATTTACTTAAGTTTTTTTCACATATATAAAATGCCCCCACTA
SwaI
TTGGCCCCCACCCGTCAGCCGAGCCGTAAGGCGAGTATTCGTTAAACGTTTTTAGAGAGAGAGAAGGAAATGAAG
GAGAGAAGGAAATGAAGGAGGAGAATAGGTAGAGTAGGTAGGGTATGGAAGAAAGAAGAGGAAGAAAGTAATGGA
ATAGAATAAATAGAATAAATAGAATAAATAAAATAAATAAAATAAAAGAGTAAAAGAAAAGAAGATGAATGGATT
AAAAGGTGTTGGAGTAGATTAAGTAGAATACGAGGATGTAGGGTAGTGCAATAGTGAAGATGGACAAAAAGTTGA
AGTTCATGGAATTAGACTATGTTGGAATTCACTAATTGTAATAAGGTGGTGTAGTGTGTATGGGTGAATAAAACG
EcoRI
GATGAAAAATTTGAAGTTGATTTGAACTGAGTGTGTTAAAGTTCATTAAGTATAATACGGTGATGTAGTGTACTA
TAGTATTTAGGATGGGTAAAAAATTGAAATGTGTGGAATTGAGTGTGCTGGAGTACGTTAAGTATAATACGGTGA
GGTAGTGTACTATAATAATTAGGATGGTTAAAAAATTTGAAGTTGTATGAATTGAGTGTGTTAGAGTTCATTAAG
TATAATACGGTGATGTAGTGTACTATAGTATTTAGGATAGGTAAAAAATTTGAAGTTGTATGAATTGAGTGTGTT
AGAGTTCATTAAGTATAATACGGTGAGGTAGTGTACTATAGTATTTAGGATGGGAAAAAATTTGAAGTTGTATGA
ATTGAGTGTGTTAGAGTTCATTAAGTATAATACGGTGAGGTAGTGTACTATAGTATTTAGGATGGGAAAAAATTG
AAATGTGTGGAATTGAGTATGGTGAAATTCACTAAGTGTAATACAGTAGTGCAGTGTATTATGATAATTAAAATG
p*ApaI* Fw
GATGAAAAATTTGAAGTTCACTCAGTCATAATTAATTGGGTAACGGAGTAACAATATAGAATAAAGGGAATTTAG
TERRA TSS **HRS**
GAAGTGCGGTAAGTTGAATAAAGAAATAGAAATGAAATACGGTATTCATAAAAAAATAAATTTACTTAAGTTTTT
TTTCACAAATACAATGCCCCCACTATTGGGCCCCACCCGTCAGCCGAGCCGTAAGGCGAGGCTGCGGGTTACAAG
ApaI pApaI Rev
GTTACGTGGTTACACGGTTACAGGTTACAGGTTACAGGGGGTTACGGTTACAGGGGTTACAGGGGTTACGGTTA
CAGGGTTACAGGTTACACGGTTACAGTTTACGGTTACAGGGTTACACGGTTACAGTTACAGGTTACAGGGTTACA
GGGGTTACGGTTACAGGTTACAGGGTTACAGGGGGGTTACAGGGGTTACAGGTTACAGGGGTTACAGGGGGTTAC
AGGGTTACACGGTTACAGGGTTAC

>pNSU70

TCAGATGCATAAAAAATTTGAAGTTGGTATGTATTGAGTGTGTTGGAGTACGGTAAGTATAATAGGGGTAAATAA

NsiI

AATGGGTAAAAAAAATTTTGAAATGTGTGGAAGTTGAGTATGTTGGAGTACATTAAGTAGATTACAGTTGTGGAG

p*NsiI* Fw

CGTACTATGGTAATGAAAATGAATGAAGAAAATGAAGTTGGGTTGAATTGAGCGTGGTAGGATTCATTATGTATG

ATTAGGAGATATAATGAGATATGGTGAATAAAAAGTTGAAATGGTGGGCTTGAGTGCGTTAGGGTGCTAGTAAGT

p*NsiI* Rev

AGAATAAAGGGGCGCAGTGTATTATGATAATTAAAATGGATGAAAAATTTGAAGTTCACTCAGTCATAATTAATT

p*ApaI* Fw

GGGTAACGGAGTAACAATATAGAATAAAGGAATTTAGGAAGTGCGGTAAGTTGAATAAAGAAATAGAAATGAAA

HRS

TACGGTATTCATAAAAAAATAAATTTACTTAAGTTTTTTTCACAAATACAATGCCCCCACTATTGGGCCCCACC

ApaI

CGTCAGCCGAGCCGTACGGCGAGTATTCGTTAAACGATTTTGGAGAGAGAGAATGGATAATGGATGGAGGTAAGA

p*ApaI* Rev

GAGGTATGAAAGAGTAGAAGATATAGAAGAGAAGAGAGAAGAAATGAGGAAGAAGTAAGATGAATAGAATAGAAG

AAACAGAGTAAGGAGAGTAAATAAATAAAGTAAGAGAGTAAAAGAAATAAGTAAAGTAAGAGGAAAGAAGATAAA

AGCAGAGGACTATATTGGAGTAGATTAAGTAGATTACAGTTGTGCAGCGTAGTATGATAATGAAGATAAAGAAAG

p*SwaI* Fw

ATAATTAAGCTGCGTTATTTATAAAAATTTAAATTTACTTAAGTTTTTTTCACATATACAATGCCCCCACTACTG

SwaI

GACCCCCACCCGTCAGCCGAGCCGTAAGGCGAGTATTCGTTAAACGTTTTTAGAGAGAGAGAAGGAAATGAAGGA

p*SwaI* Rev

GAGAAGGAAATGAAGGAGAGAAGGAAATGAAGGAGGAGAATAGGTAGAGTAGGTAGGTAGAGTAGGTAGAGTAGG

TAGAGAGTAGGTAGGATAGAGTATGGAAGAAAGAAGAGGAAGAAAGTAATGGAATAGAATAAATAGAATAGAATA

AATAGAATAGAATAAATAGAATAAATAGAATAAATAAAATAAAAGAGTAAAAGAAAAGAAGATGAATGGATTAAA

AGGTGTTGGAGTAGATTAAGTAGAATACGAGGATGTAGGGTAGTGCAATAGTGAAGATGGACAAAAAGTTGAAGT

TCATGGAATTAGACTATGTTGGAATTCACTAATTGTAATAAGGTGGTGTAGTGTGTATGGGTGAATAAAACGGAT

EcoRI

GAAAAATTTGAAGTTGATTTGAACTGAGTGTGTTAAAGTTCATTAAGTATAATACGGTGATGTAGTGTACTATAG

TATTTAGGATGGGTAAAAAATTGAAATGTGTGGAATTGAGTGTGCTGGAGTACGTTAAGTATAATACGGTGAGGT

AGTGTACTATAATAATTAGGATGGTTAAAAAATTTGAAGTTGTATGAATTGAGTGTGTTAGAGTTCATTAAGTAT

AATACGGTGATGTAGTGTACTATAGTATTTAGGATAGGTAAAAAATTTGAAGTTGTATGAATTGAGTGTGTTAGA

GTTCATTAAGTATAATACGGTGAGGTAGTGTACTATAGTATTTAGGATGGGAAAAAATTTGAAGTTGTATGAATT

GAGTGTGTTAGAGTTCATTAAGTATAATACGGTGAGGTAGTGTACTATAGTATTTAGGATGGGAAAAAATTGAAA

TGTGTGGAATTGAGTATGGTGAAATTCACTAAGTGTAATACAGTAGTGCAGTGTATTATGATAATTAAAATGGAT

pApaI Fw

GAAAAATTTGAAGTTCACTCAGTCATAATTAATTGGGTAACGGAGTAACAATATAGAATAAAGGAATTTAGGAA

TERRA TSS **HRS**

GTGCGGTAAGTTGAATAAAGAAATAGAAATGAAATACGGTATTCATAAAAAAATAAATTTACTTAAGTTTTTTT

CACAAATACAATGCCCCCACTATTGGGCCCCACCCGTCAGCCGAGCCGTAAGGCGAGGCTGCGGGTTACAAGGTT

ApaI pApaI Rev

ACGTGGTTACACGGTTACAGGTTACAGGTTACAGGGGGTTACGGTTACAGGGGTTACAGGGGTTACGGTTACAG

GGTTACAGGTTACACGGTTACAGTTTACGGTTACAGGGTTACACGGTTACAGGGTTACAGGGGTTACGGTTACAC

GGTTACAGGGGGTTACAGGGTTACAGGGGTTACAGGTTACAGGGGGTTACAGGGGTACGGTTACAGGGGGGGGTT

ACGGTTACAGGGGTTACAGG

Supplementary Figure 2. **Subtelomeric region sequences of pNSU70 and pNSU64.**
The homologous repeated sequence (HRS, grey box), colored oliogonucleotides used for RT-qPCR, and restriction sites are annotated in subtelomeric sequences of pNSU64 and pNSU70.

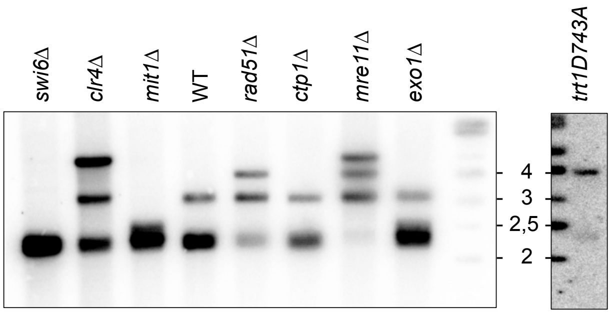

Supplementary Figure 3. **Subtelomeric patterns of strains used in this study.**
Genomic DNA of the indicated strains was digested by *Nsi*I and analyzed by Southern blot with a STE1 probe.

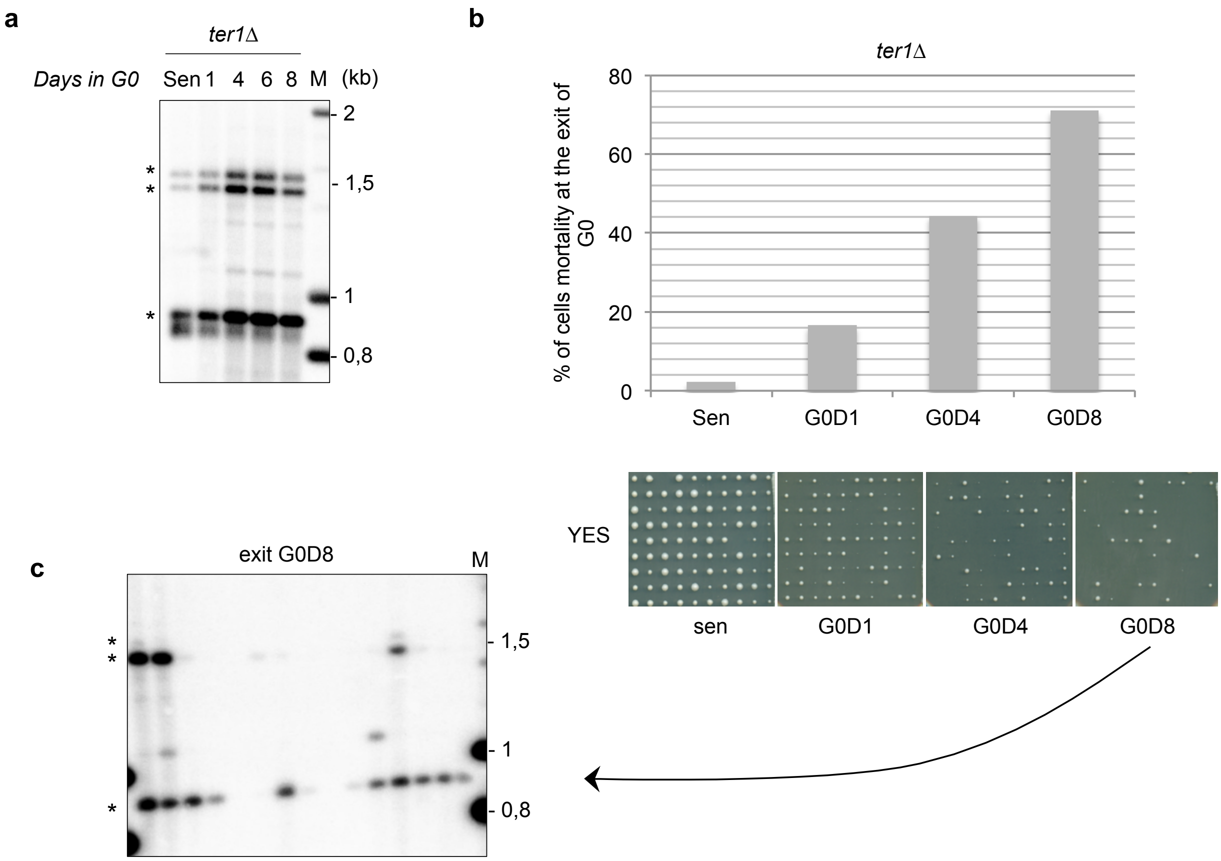

Supplementary Figure 4. Cell viability at G0 exit.

a) *EcoR*I-digested genomic DNAs from senescent and nitrogen-starved *ter1*D cells were analyzed by Southern blot.

b) Senescent (Sen) and quiescent *ter1*D cells after 1, 4, or 8 days in G0 were micromanipulated and plated onto YES plate. The percentage of cells that were not able to form a colony was plotted. Pictures of agar plates are shown

c) Fifteen clones that were able to exit G0 after 8 days of quiescence were grown for approximately 32 pds. Their genomic DNA was then extracted, digested by *EcoR*I and analyzed by Southern blot with a Telo/STE1 probe. STEEx are shown by asterisks.
